# Supplementary material for: Enzymatic Biotransformation of Balloon Flower Root Saponins into Bioactive Platycodin D by Deglucosylation with Caldicellulosiruptor bescii β-Glucosidase
Source: Int J Mol Sci. 2019 Aug 7;20(16):3854. doi: 10.3390/ijms20163854 (PMC6721153; doi:10.3390/ijms20163854)
Supplement: Supplementary file 1 [file ijms-20-03854-s001.pdf]

Supplemental Figures

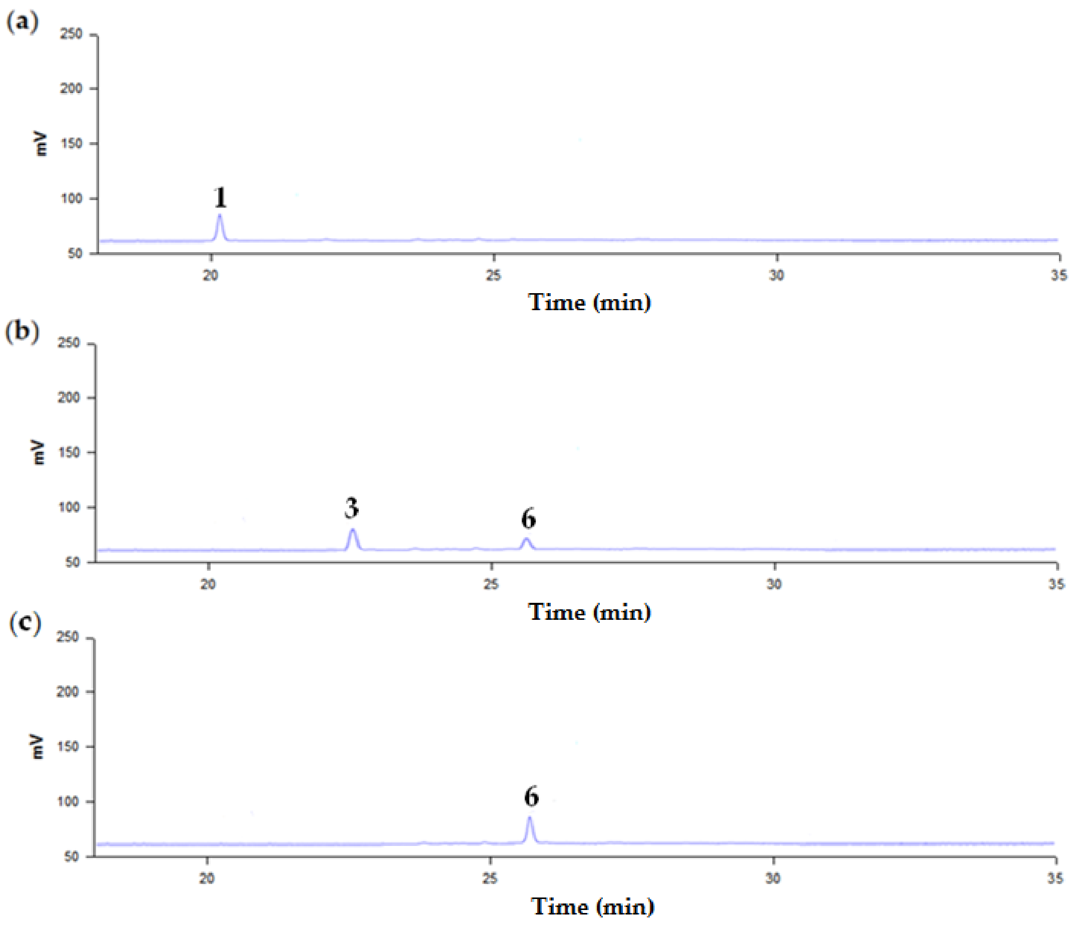

Figure S1. HPLC profiles showing the change of deapiosylated (deapi)-platycoside E (Deapi-PE) (1) via deapi-platycodin D3 (3) to deapi-platycodin D (6). Deapi-PE was reacted with β-glucosidase from *Caldicellulosiruptor bescii* for (A) 0 h, (B) 0.2 h, and (C) 2 h.

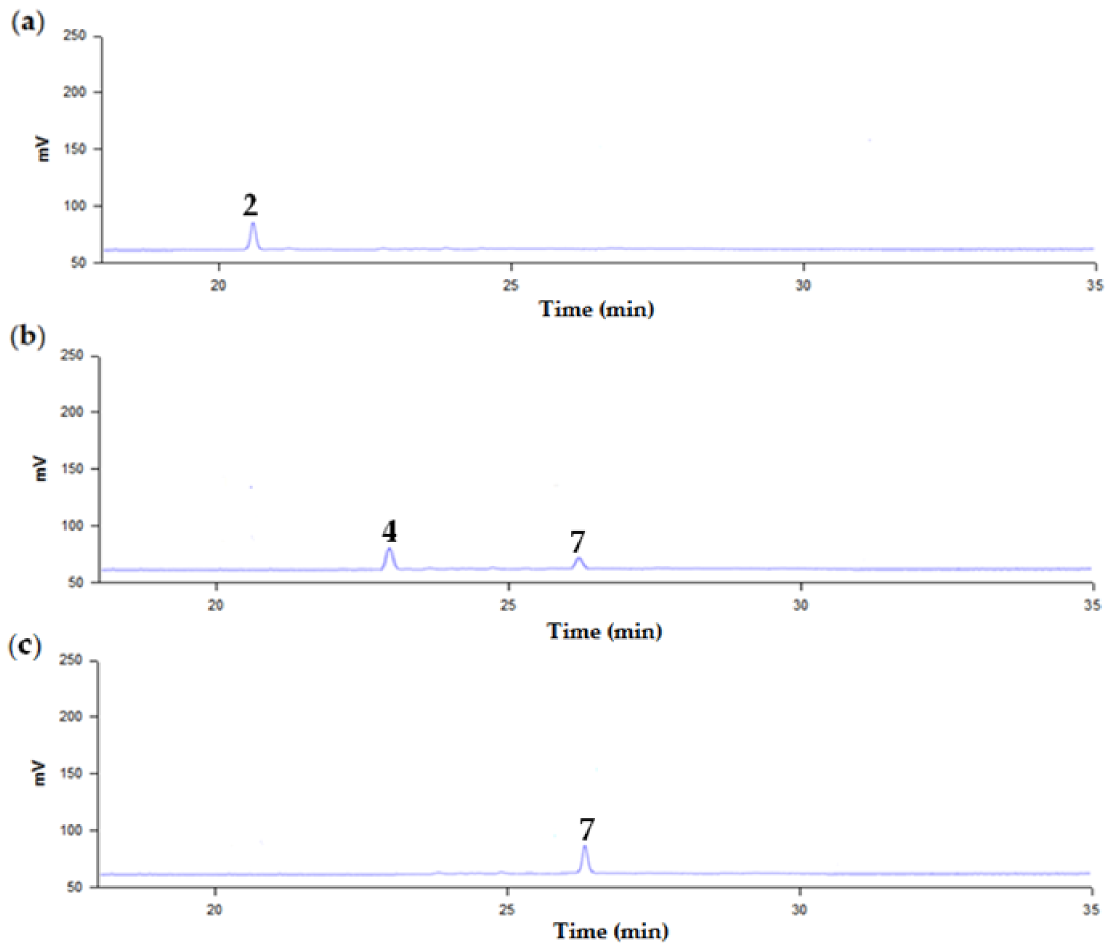

Figure S2. HPLC profiles showing the change of platycoside E (PE) (2), via platycodin D3 (4), to platycodin D (7). PE was reacted with β-glucosidase from *Caldicellulosiruptor bescii* for (A) 0 h, (B) 0.2 h, and (C) 2 h.

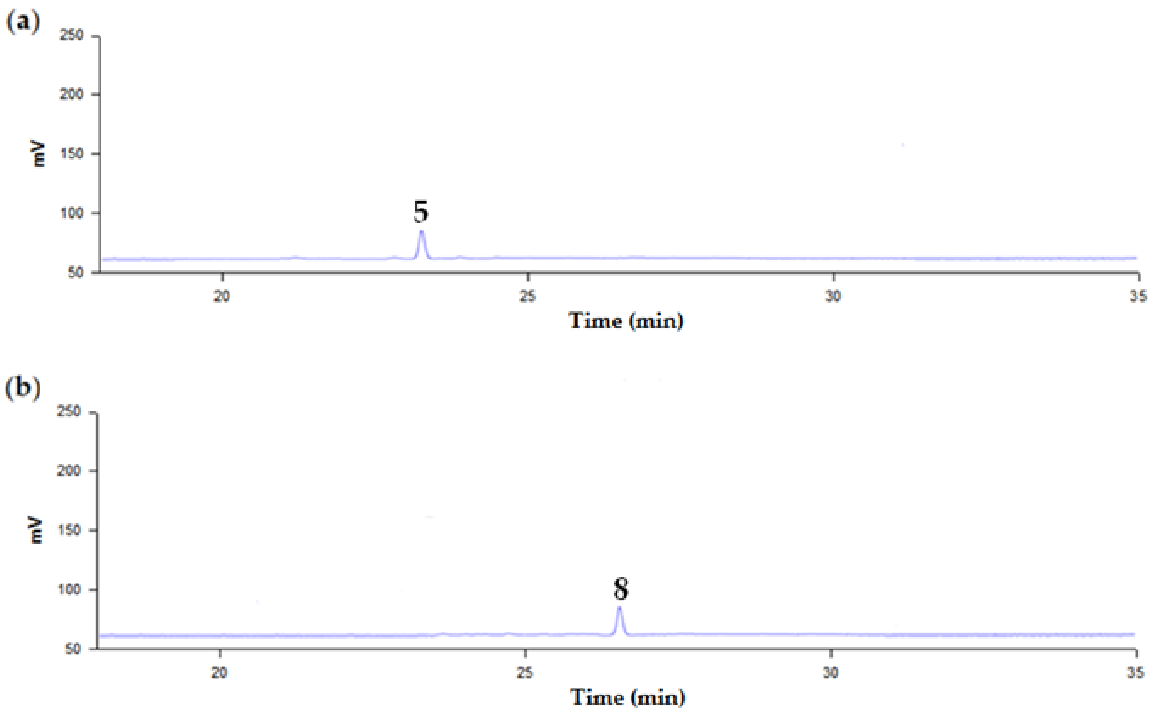

Figure S3. HPLC profiles showing the change of polygalacin D3 (5) to polygalacin D (8). Polygalacin D3 was reacted with β-glucosidase from *Caldicellulosiruptor bescii* for (A) 0 h and (B) 2 h.

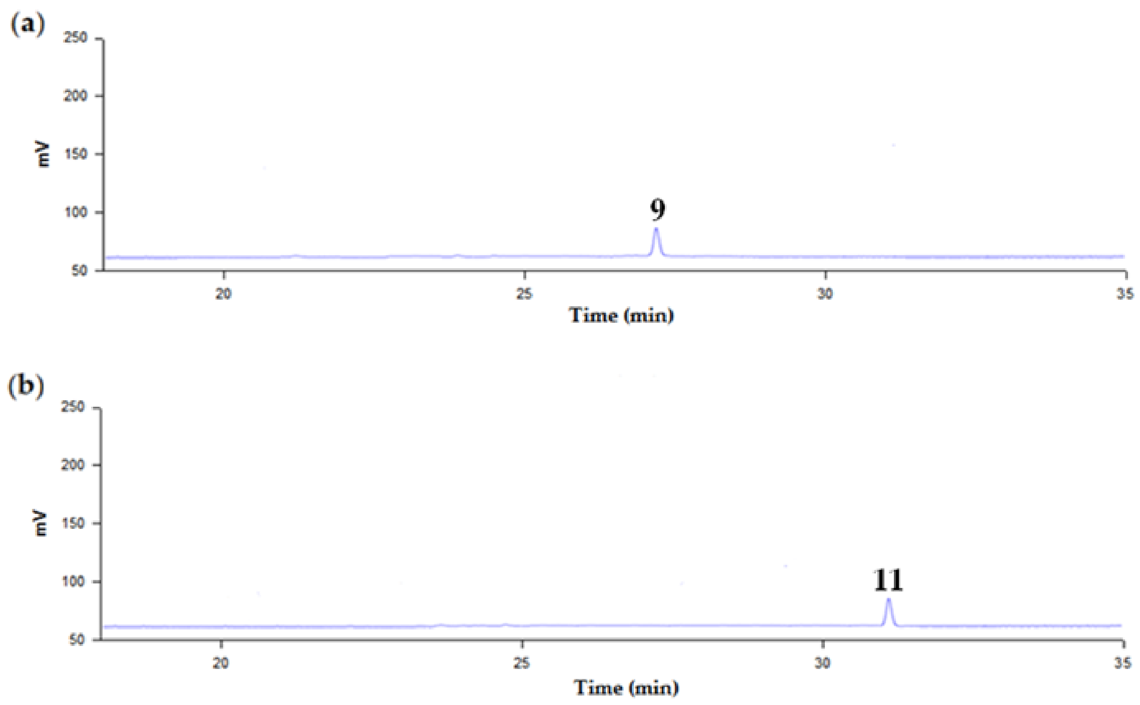

Figure S4. HPLC profiles showing the change of 3″-O-acetyl polygalacin D3 (9) to 3″-O-acetyl polygalacin D (11). 3″-O-Acetyl polygalacin D3 was reacted with β-glucosidase from *Caldicellulosiruptor bescii* for (A) 0 h and (B) 2 h.

(**a**)

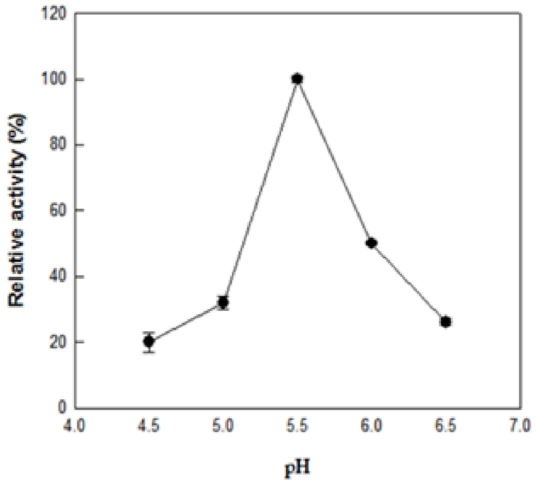

(**b**)

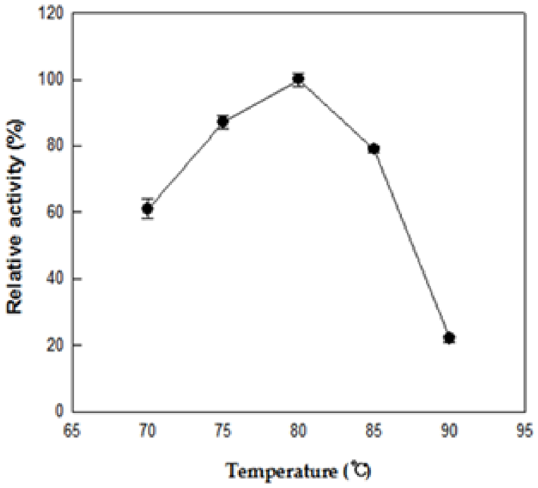

Figure S5. Effects of pH and temperature on the production of platycodin D (PD) from platycoside E (PE) by β-glucosidase from *Caldicellulosiruptor bescii*. (A) pH effect. The reactions were performed in 50 mM citrate/phosphate buffer (pH 5.5) containing 0.05 mg/mL enzyme and 0.4 mg/ml PE, at 80 °C for 10 min. (B) Temperature effect. The reactions were performed in 50 mM citrate/phosphate buffer (pH 5.5) containing 0.05 mg/mL enzyme and 0.4 mg/mL PE, by varying the temperature from 70 to 90 °C for 10 min. Data are the means of three experiments, and error bars represent the standard deviation.

(**a**)

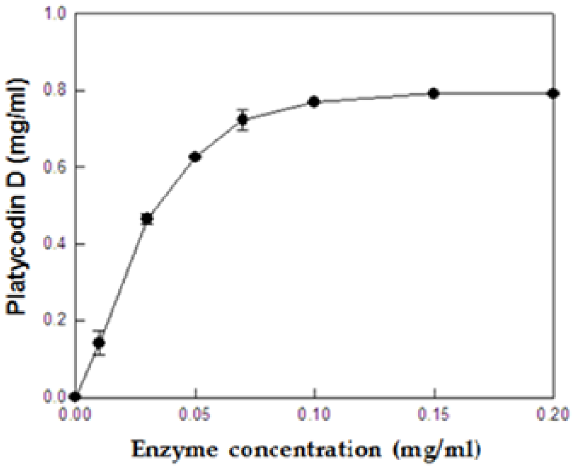

(**b**)

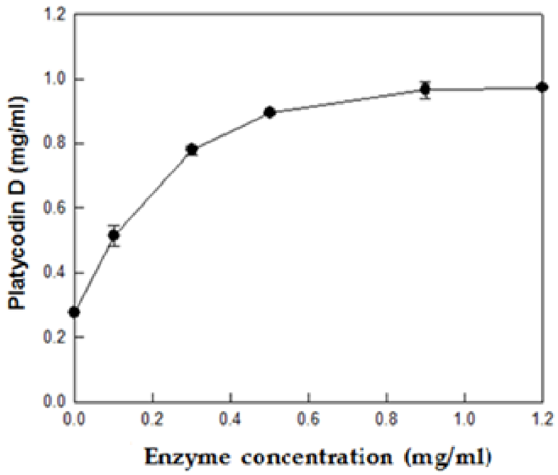

Figure S6. Effects of enzyme concentration on the production of platycodin D (PD) from platycoside E (PE) by β-glucosidase from *Caldicellulosiruptor bescii*. (A) Reagent-grade PE. (B) PE in Platycodi radix extract. The reactions were performed for 30 min at 80 °C in 50 mM citrate/phosphate buffer (pH 5.5) containing 1 mg/mL PE and 0.07 mg/mL enzyme or 1 mg/mL PE and 0.5 mg/mL enzyme. Data are the means of three experiments, and error bars represent the standard deviation.
